# High-quality GeSn Layer with Sn Composition up to 7% Grown by Low-Temperature Magnetron Sputtering for Optoelectronic Application

**DOI:** 10.3390/ma12172662

**Published:** 2019-08-21

**Authors:** Jiayin Yang, Huiyong Hu, Yuanhao Miao, Linpeng Dong, Bin Wang, Wei Wang, Han Su, Rongxi Xuan, Heming Zhang

**Affiliations:** 1 Key Laboratory of Wide-Gap Semiconductor Materials and Devices, School of Microelectronics, Xidian University, Xi’an 710071, China; 2 Guangdong Provincial Key Laboratory of Optical Fiber Sensing and Communications, Jinan University, Guangzhou 510632, China; 3 Department of Optoelectronic Engineering, Jinan University, Guangzhou 510632, China; 4 School of Physics and Electronic Information, Yunnan Normal University, Kunming 650500, China; 5 Yunnan Key Laboratory of Opto-electronic Information Technology, Kunming 650500, China

**Keywords:** GeSn layer, averaged TDD, magnetron sputtering, p-i-n diode

## Abstract

In this paper, a high-quality sputtered-GeSn layer on Ge (100) with a Sn composition up to 7% was demonstrated. The crystallinity of the GeSn layer was investigated via high-resolution X-ray diffraction (HR-XRD) and the strain relaxation degree of the GeSn layer was evaluated to be approximately 50%. A novel method was also proposed to evaluate the averaged threading dislocation densities (TDDs) in the GeSn layer, which was obtained from the rocking curve of GeSn layer along the (004) plane. The photoluminescence (PL) measurement result shows the significant optical emission (1870 nm) from the deposited high-quality GeSn layer. To verify whether our deposited GeSn can be used for optoelectronic devices, we fabricated the simple vertical p-i-n diode, and the room temperature current–voltage (I–V) characteristic was obtained. Our work paves the way for future sputtered-GeSn optimization, which is critical for optoelectronic applications.

## 1. Introduction

Recently, GeSn alloys have been shown the capability to become a real direct bandgap material with a Sn composition of 10% [1,2,3], which makes it a promising optical gain medium for group IV light sources [4,5,6]. Moreover, GeSn is also an attractive material owing to its compatibility with the mature Si complementary metal-oxide-semiconductor (CMOS) process. The epitaxial growth of high-Sn-composition GeSn with high material quality is challenging because the solid solubility of Sn in Ge or Ge in Sn is less than 1%. The smaller surface free energy of Sn compared to that of Ge makes Sn more likely to immigrate to the surface of the GeSn film during epitaxial growth and thermal treatment [7,8,9,10,11]. So far, several techniques such as chemical vapor deposition (CVD) [12,13,14], molecular beam epitaxy (MBE) [15,16,17], and magnetron sputtering [18,19,20,21,22] have been employed to achieve the crystalline GeSn layers.

Reduced pressure chemical vapor deposition (RPCVD) has grown high-quality GeSn with a Sn composition up to 12.6% using Ge_2_H_6_ and SnCl_4_ as precursors, thus achieving the first demonstration of an optically pumped GeSn laser [23]. Lasing from GeSn micro disks with a Sn composition up to 16% has also been achieved by using the same precursors [24]. At the same time, GeH_4_ and SnCl_4_ have been utilized to grow high-quality GeSn with a Sn composition up to 17%, also contributing to the achievement of a GeSn laser with a wavelength ranging from 2 to 3 µm and a maximum lasing temperature of 180 K [25]. Besides, high-order Ge hydrides such as Ge_3_H_8_ and Ge_4_H_10_ were also utilized to pursue high-Sn-content GeSn layers [26,27,28]. High-quality GeSn thin film with a high Sn content (22.3%) was deposited on Sn-graded GeSn buffer. Optically pumped lasing has also been observed at a wavelength of 3 µm and lasing temperatures of 150 and 180 K [29,30,31]. SiGeSn/GeSn/SiGeSn quantum well (QW) laser on Si with a Sn composition up to 14.4% has also been achieved, which demonstrated a maximum operation temperature of 90 K and its minimum lasing threshold was only 25 kW/cm^2^ [32]. MBE has grown high-quality GeSn, which contributes to the demonstration of GeSn light-emitting diodes (LEDs) [4,33,34,35] and photodetectors [36,37,38]. However, a GeSn laser with the GeSn layer deposited by MBE has not been reported yet, which is mainly due to the lower Sn composition.

Comparing CVD and MBE, magnetron sputtering is also an alternative method to grow GeSn alloys. So far, only a few groups have grown crystalline GeSn alloys. A high-quality GeSn layer was grown on a Ge (100) substrate by magnetron sputtering, which contributed to the first demonstration of GeSn p-i-n photodetectors [18]. A high-quality SiGeSn/GeSn multiple quantum well (MQW) structure was also grown on a Ge substrate by sputtering epitaxy, and the Sn content of the GeSn layer was 6%, which contributed to the demonstration of GeSn photodetector [22]. In addition, crystalline GeSn thin films with a high Sn content (28%) were also deposited on low-Sn-composition GeSn buffer layers using sputtering epitaxy [20]. Our previous work showed the deposition of GeSn on a tensile-strained Ge buffer with a low Sn content (3%), and the room temperature photoluminescence spectrum was observed [21]. However, no room temperature photoluminescence spectrum or GeSn-based diode were reported for a higher Sn composition GeSn alloy deposited by this method. In this paper, we propose a novel method to obtain the threading dislocation densities TDDs of GeSn layers from GeSn (004) rocking curves.

In this work, we grew a high-quality GeSn layer on a Ge (100) substrate with a Sn composition up to 7%. Room temperature optical emission from the GeSn layer was observed and the averaged TDD in the layer was also obtained from the rocking curve along the (004) plane. To further verify the material quality of the film, we fabricated a simple vertical GeSn p-i-n diode.

## 2. Methods

### 2.1. Preparation of GeSn Layers

In our material growth experiment, a physical vapor deposition (PVD) system manufactured by Kurt J. Lesker Corporation (Shanghai, China) was utilized to deposite the GeSn layer. A high pure Ge target (99.999%) and Sn target (99.999%) were employed to deposit the GeSn layer on a Ge substrate. The diameter and the thickness of the targets were 50.8 and 0.25 mm, respectively. To prevent excess heating, the Ge target and Sn target were bonded to a copper baking plate. The GeSn layer with a Sn content up to 7% was deposited directly onto the Ge (100) substrate by magnetron co-sputtering, and the Ge wafer was n-doped. At first, running deionized water was utilized to clean the Ge wafer, which was then etched by HF. Due to the existence of a thin oxide layer on the Ge substrate, we dipped a mixture of H_2_O_2_ and H_2_O for a few seconds and the thin oxide layer was dislodged by dipping in HF. This procedure was performed several times to ensure the removal of several atomic layers of Ge. After cleaning, the Ge wafer was loaded into the growth chamber and the base pressure for the chamber was set to 10^−6^ mTorr. Then, the Ge substrate was warmed to be 160 °C prior to the growth of the GeSn film. During the progress for the growth of GeSn, the pressure was maintained at 3 mTorr via argon. We controlled the composition of Ge and Sn in the film by controlling the RF power of 120 W for the Ge target and changing the RF power of the Sn target. The growth rate was around 0.2–0.5 nm/s, and the evaluated thickness for GeSn layer was around 400 nm.

### 2.2. Fabrication of Simple GeSn Diode

**Figure 1:**
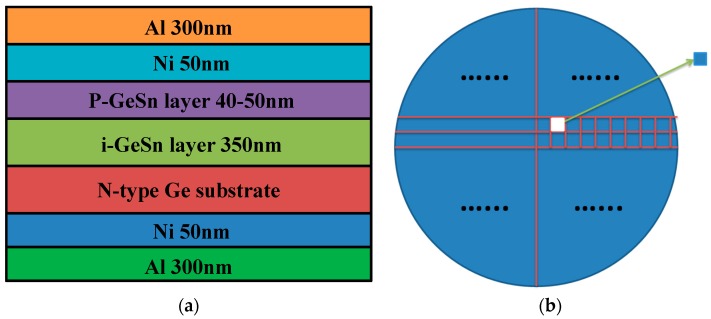
(**a**) The cross-section image for the layer structure of the sample, (**b**) dicing diagram of the sample.

After the deposition of the GeSn layer, a B implant (dose of 4 × 10^15^ cm^−2^ and energy of 40 keV) was performed to dope the 400 nm i-GeSn, followed by rapid thermal annealing (RTA) in N_2_ to activate the p-type dopant concentration. Then, the p-i-n diode was processed via metallization and RTA treatment. The schematic cross-section of the layer structure of the sample is shown in Figure 1a. In order to form a simple GeSn p-i-n diode, we cut the sample into small pieces of 0.8 × 0.8 mm square by laser slicing. Then, the typical current–voltage (I–V) characteristic of the sample was measured by a Keithley 4200 semiconductor characterization system parameter analyzer (Tektronix, Beaverton, OR, USA).

### 2.3. Characterization Method for GeSn Layer

The crystallinity quality of the GeSn layer was determined by high-resolution X-ray diffraction (HR-XRD) measurement. The Sn composition of the GeSn layer was verified by energy dispersive spectrometry (EDS). The XRD 2θ-ω scans along the (004) and (224) planes were accomplished. The rocking curve of GeSn (004) was also found to determine the averaged threading dislocation densities (TDDs) in the GeSn layer. PL was used to ascertain the band gap energy. The photoluminescence (PL) setup consisted of a Fourier transform infrared spectroscopy, liquid nitrogen (LN_2_)-cooled InGaAs detector, a 532 nm continuous wave (CW) laser, and a grating monochromator.

## 3. Results and Discussion

### 3.1. Crystallinity, Sn Content, and Strain of GeSn Layer

**Figure 2:**
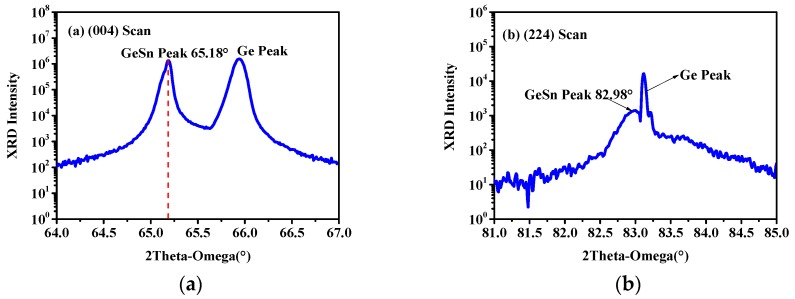
High-resolution 2θ-ω X-ray diffraction (XRD) scans of the GeSn alloy along (**a**) the (004) plane, (**b**) the (224) plane.

Figure 2a shows the XRD 2θ-ω (004) scan of the sample, in which the diffraction peak of GeSn and Ge can be seen. The peaks at 66° and 65.18° correspond to the Ge substrate and GeSn layer, respectively. The out-of-plane lattice constant of the GeSn layer was extracted from the following expression:

(1) $$a_{Ge_{1-x}Sn_{x}}^{⊥}=\frac{2\lambda }{\sin\theta_{004}}$$

where *λ* represents the wavelength of Cu K1 (*λ* = 1.5406 nm) and *θ*_004_ is the diffraction peak of the GeSn layer along the (004) plane. Hence, the out-of-plane lattice constant of the GeSn layer is calculated to be 0.5721 nm. Figure 2b shows the XRD 2θ-ω (224) scan of the sample in which the diffraction peaks of GeSn and Ge can also be clearly seen. The peaks at 83.1° and 82.98° correspond to the Ge substrate and the GeSn layer, respectively. The in-plane lattice constant of the GeSn layer can be reckoned as:

(2) $$a_{Ge_{1-x}Sn_{x}}^{\left|\right|}={\left[\frac{8}{\frac{1}{d_{224}^{2}}-\frac{16}{{a_{Ge_{1-x}Sn_{x}}^{⊥}}^{2}}}\right]}^{1/2}$$

where *d*_224_ is the crystal spacing. For the XRD scan along the (224) plane, *d*_224_ can be calculated using the following equation:

(3) $$d_{hkl}=\frac{\lambda }{2\sin\theta_{hkl}}.$$

Therefore, *d*_224_ of the GeSn layer is estimated to be 0.1162 nm. Substituting the value of *d*_224_ into Equation (2), the in-plane lattice constant of the GeSn layer is calculated to be 0.5634 nm.

**Figure 3:**
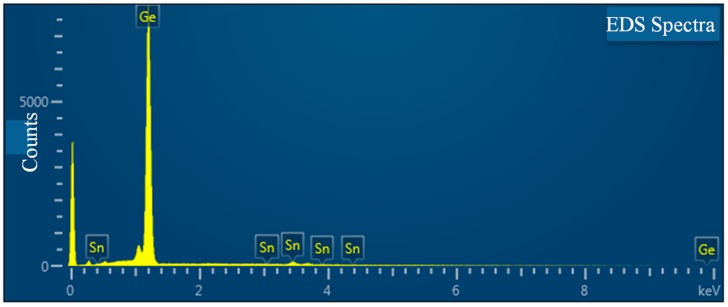
Surface energy dispersive spectrometry (EDS) spectra of the GeSn layer.

To confirm the Sn composition for the GeSn layer, we performed the surface EDS spectra of the GeSn layer. The spectra image was obtained from typical EDS analysis. Figure 3 shows that seven primary peaks were formed at 0.4, 1.3, 3, 3.4, 3.6, 4.4, and 9.86 keV. The peaks at 0.4, 1.3, 3, 3.6 and 4.4 keV match the spectral lines of Sn. Furthermore, the peaks at 1.3 and 9.86 keV match the spectral lines of Ge. Thus, the Sn composition of the GeSn layer is estimated to be 7%. Based on the lattice elastic theory, the bulk lattice constant of GeSn ($a_{bulk}$) can be obtained using the following equation:

(4) $$\frac{a_{⊥}-a_{bulk}}{a_{//}-a_{bulk}}=-\frac{{2C}_{12}}{C_{11}}$$

where $a_{⊥}$ is the out-of-plane lattice constant of the GeSn layer, $a_{//}$ is the in-plane lattice constant of the GeSn layer, C_11_ and C_12_ are the elastic coefficients. Moreover, there is a relationship between C_11_, C_12_, and the Sn composition of the GeSn [39]:

(5) $$\frac{C_{11}}{C_{12}}=0.3738+0.1676x-0.0296x^{2}.$$

We can first substitute the Sn composition (7) obtained from EDS into Equation (5), and the value of C_11_/C_12_ is calculated to be 0.0966. $a_{bulk}$ can also be calculated using the in-plane lattice constant of the GeSn layer, the out-of-plane lattice constant of the GeSn layer, and the value of C_11_/C_12_ (0.0966). The bulk lattice constant of the GeSn is calculated to be 0.5707 nm.

Therefore, the relaxation degree can be demonstrated as:

(6) $$R=\frac{a_{//}^{\mathrm{GeSn}}-a_{⊥}^{GeSn}}{a_{bulk}^{GeSn}-a^{Ge}}$$

where $a^{Ge}$ is the lattice constant of the Ge ($a^{Ge}$ = 0.5658 Å), $a_{⊥}$ is the out-of-plane lattice constant of the GeSn, $a_{//}$ is the in-plane lattice constant of the GeSn, and $a_{bulk}$ is the bulk lattice constant of the GeSn. Finally, the relaxation degree of the GeSn layer is evaluated to be approximately 50%.

**Figure 4:**
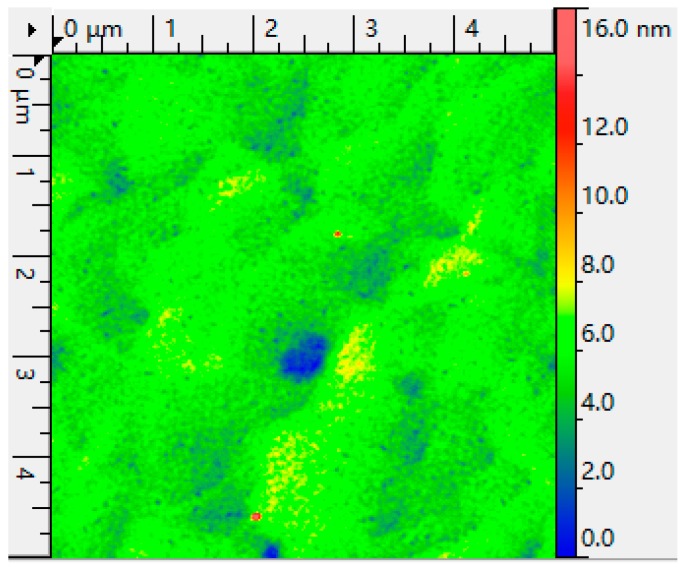
A 5 μm × 5 μm atomic force microscopy (AFM) image of the GeSn layer deposited by magnetron sputtering.

In order to determine the surface morphology of the GeSn layer, atomic force microscopy (AFM) measurement was performed. Figure 4 shows the typical 5 μm × 5 μm AFM image of the GeSn layer and the RMS (root mean square roughness) value of the GeSn sample was extracted from AFM scans. The as-grown GeSn layer showed a smooth surface, and the root mean square roughness (Rq) value and average root mean square (Ra) for the GeSn sample were found to be 0.8 and 0.6 nm, respectively. Compared with other low-Sn-composition GeSn layer deposited by magnetron sputtering (Table 1), our result is better than other RMS values of the crystalline GeSn layer.

### 3.2. Averaged TDD in GeSn Layer

Transmission electron microscopy (TEM) and etching pit density (EPD) are utilized to evaluate the TDDs in GeSn layers. Meanwhile, it is very difficult to ascertain the TDDs for GeSn using EPD when the TDDs are beyond 10^6^ cm^−2^. Moreover, TEM is also limited by its small affected region because TEM can only obtain the TDDs of the material in a small region. So, we evaluated the TDDs of the GeSn layer using the rocking curve. From our previous report [41], the intrinsic FWHM (full width at half maxima) for the strained GeSn can be described as:

(7) $$\beta_{0}(\mathrm{hkl})=[r_{c}\lambda^{2}(1+|\cos2\theta |)|F_{hkl}|]\times {\left[\sin(\theta -\phi )/\sin(\theta +\phi )\right]}^{1/2}/[\pi a_{0}^{3}\sin(2\theta )]$$

where r_c_ is the radius of the electron, *θ* is the Bragg angle for the GeSn, *λ* is the X-ray wavelength, $\left|F_{hkl}\right|$ is the reflection structure factor of the GeSn (hkl), $a_{0}$ is the lattice constant of the bulk GeSn, and *ϕ* is the angle between the crystal surface and the diffracting planes. The reflection structure factors for the GeSn (004) are all 64f^2^, where f is the dispersion factor for the atom.

**Figure 5:**
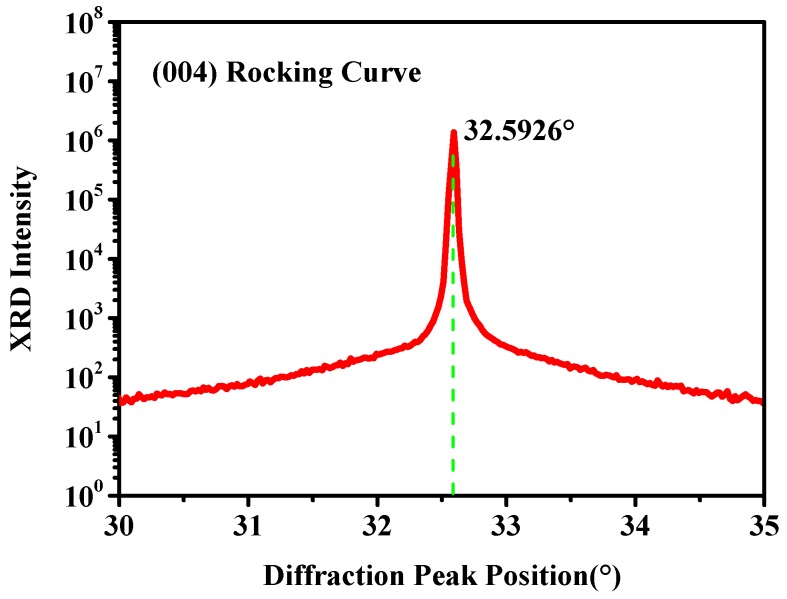
Rocking curve of the GeSn layer along the (004) plane.

The FWHM broadening by the TDDs in GeSn layer can represented as:

(8) $$\beta_{TDD}^{2}\approx \beta_{m}^{2}(\mathrm{hkl})-\beta_{0}^{2}(\mathrm{hkl})-\beta_{d}^{2}(\mathrm{hkl})$$

where $\beta_{m}$ is the measured FWHM of the strained GeSn, $\beta_{0}$ is the intrinsic FWHM of the strained GeSn, and $\beta_{d}$ is the FWHM broadening by incident beam difference of the HR-XRD equipment. Owing to the large lattice constant mismatch between Ge and GeSn, the majority of the dislocation locates at the interface between Ge and GeSn. The TDDs in GeSn decreased with the increase of thickness and the previously deposited GeSn layer can be regarded as a buffer layer for the top GeSn layer. So, we believe that the TDDs in the whole GeSn layer are not uniform. However, the XRD rocking curve measurement (Figure 5) demonstrates that the FWHM of the whole GeSn layer and the TDDs obtained from the XRD result reflect the TDDs from the whole GeSn layer. For this reason, we can conclude that the TDDs obtained from the XRD result can be used to present the averaged TDD of the whole GeSn layer.

The averaged TDD in the strained GeSn layer can be expressed as an empirical formula:

(9) $$D=\beta_{TDD}^{2}/2\pi b^{2}\ln2.$$

The value of b is 0.4. The calculated values of $\beta_{m}^{2}$, $\beta_{0}^{2}$, $\beta_{d}^{2}$, $\beta_{TDD}^{2}$, and the TDDs of the GeSn layer are outlined in Table 2. In addition, we compare the calculated TDDs with other results, and it was found that the calculated value agrees well with the reference TDDs.

**Figure 6:**
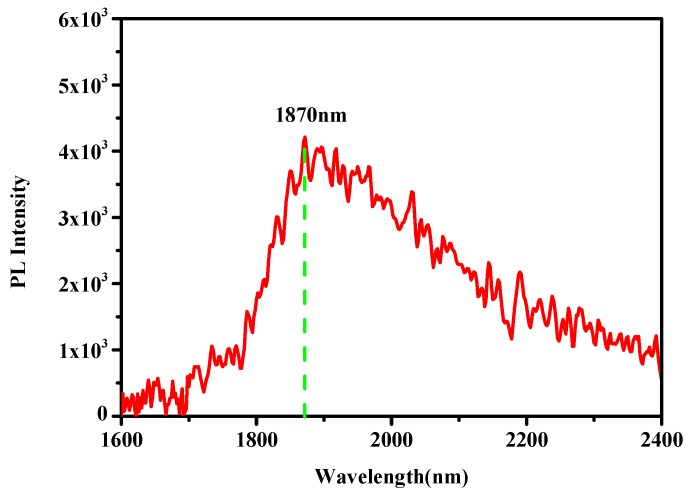
Room temperature photoluminescence (PL) spectra of the GeSn layer deposited by magnetron sputtering.

The photoluminescence (PL) characterization was undertaken to examine the luminescence property of the GeSn layer deposited by magnetron sputtering. The PL measurement was excited by a 532 nm green laser. During the excitation, laser beam was focused to be 100 µm spot and its power was intercalated as 500 mW. The PL emission was collected by FTIR analysis, equipped with a liquid nitrogen-cooled (LN_2_) InGaAs detector. Figure 6 shows the room temperature PL spectra of the GeSn layer and the luminescence peak was located at 1870 nm. Comparing the PL peak position of GeSn with the results of another work [1], our result is smaller than that of a direct bandgap GeSn with a 2230 nm PL peak. From the HR-XRD result, the relaxation degree of the GeSn layer was evaluated to be approximately 50%. Therefore, we conclude that the GeSn layer is an indirect bandgap material. This finding can be attributed to the large lattice mismatch between Ge and GeSn (the lattice constant of GeSn is larger than Ge), which indicates that GeSn layer is under compressive strain and it is adverse to the bandgap transformation of Ge from indirect GeSn material to direct GeSn material.

**Figure 7:**
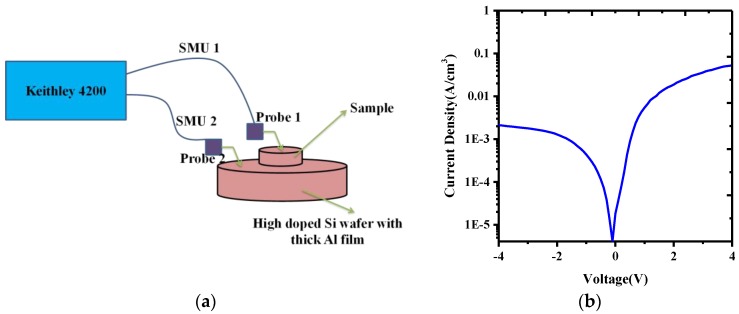
(**a**) Current–voltage (I–V) characteristic setup, (**b**) room temperature current–voltage (I–V) characteristic of an 8 mm × 8 mm p-i-n diode with the GeSn layer deposited by magnetron sputtering.

Finally, the current–voltage (I–V) characteristic was carried out at room temperature. The electrical property of the p-i-n diode was performed using a Keithley 4200 semiconductor characterization system parameter analyzer (Figure 7a). Figure 7b shows the typical I–V characteristic of an 8 mm × 8 mm square p-i-n diode with the GeSn layer deposited by magnetron sputtering. The very high dynamic resistance may be attributed mainly to the fact that the Si wafer and Ge substrate have a high series resistor. Ultimately, we conclude that the sputter-deposited GeSn layer has the great potential to be used for the fabrication of optoelectronic devices.

## 4. Conclusions

In summary, a high-quality GeSn layer with a Sn content up to 7% was successfully grown on a Ge (100) substrate via magnetron co-sputtering. The HR-XRD result shows the remarkable single-crystalline property of the GeSn layer. Due to the fact that the TDDs in the whole GeSn layer are not uniform, we conclude that the TDDs obtained from the XRD result can give the averaged TDD of the whole GeSn layer (8.7 × 10^9^ cm^−2^) along the (004) plane. The PL measurement result shows the optical emission from the deposited high-quality GeSn layer. Furthermore, the fabricated vertical p-i-n device exhibits a good room temperature current–voltage (I–V) characteristic. From the results, we predict that the sputter-deposited GeSn will have great potential to achieve high-Sn-composition GeSn layers with proper design, which is critical for optoelectronic applications.

## Figures and Tables

**Table 1:** Comparison of the RMS (root mean square roughness) values of crystalline GeSn with those found in other references.

Reference	Scanned Area (μm × μm)	RMS Value (nm)
[18]	10 × 10	1.033–6.982
[40]	4 × 4	0.88
This work	5 × 5	0.8

**Table 2:** Comparison of the calculated values of $\beta_{m}^{2}$, $\beta_{0}^{2}$, $\beta_{d}^{2}$, $\beta_{TDD}^{2}$, threading dislocation densities (TDDs), and reference TDDs of GeSn.

Reference	FWHM (arcsec)	$\beta_{m}^{2}$ (arcsec)^2^	$\beta_{0}^{2}$ (arcsec)^2^	$\beta_{d}^{2}$ (arcsec)^2^	$\beta_{TDD}^{2}$ (arcsec)^2^	Calculated TDDs (cm^−2^)	Ref TDDs (cm^−2^)
This work	627	395,641	1324.32	419.068	393,897.702	1.89 × 10^9^	-
[41]	2056	4,227,136	1324.32	419.068	4,225,392.61	2.03 × 10^10^	2 × 10^10^
[42]	197	38,809	134.32	419.068	38,065.12	1.78 × 10^8^	6.58 × 10^7^
